# Supplementary figures and images for: Detection of hard and soft selective sweeps from Drosophila melanogaster population genomic data
Source: PLoS Genet. 2021 Feb 26;17(2):e1009373. doi: 10.1371/journal.pgen.1009373 (PMC7946363; doi:10.1371/journal.pgen.1009373)

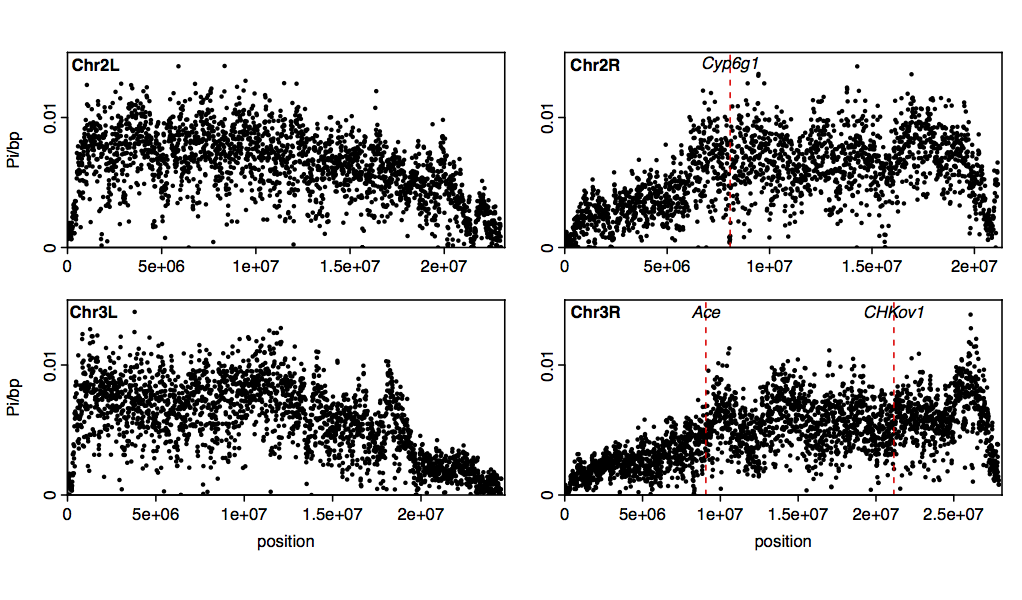

Supplement: S1 Fig — Each point represents the mean Pi/bp value in a 10Kb window. Red vertical lines indicate the positions of the positive controls, Ace, Cyp6g1, and CHKov1. (TIF) [file pgen.1009373.s001.tif]

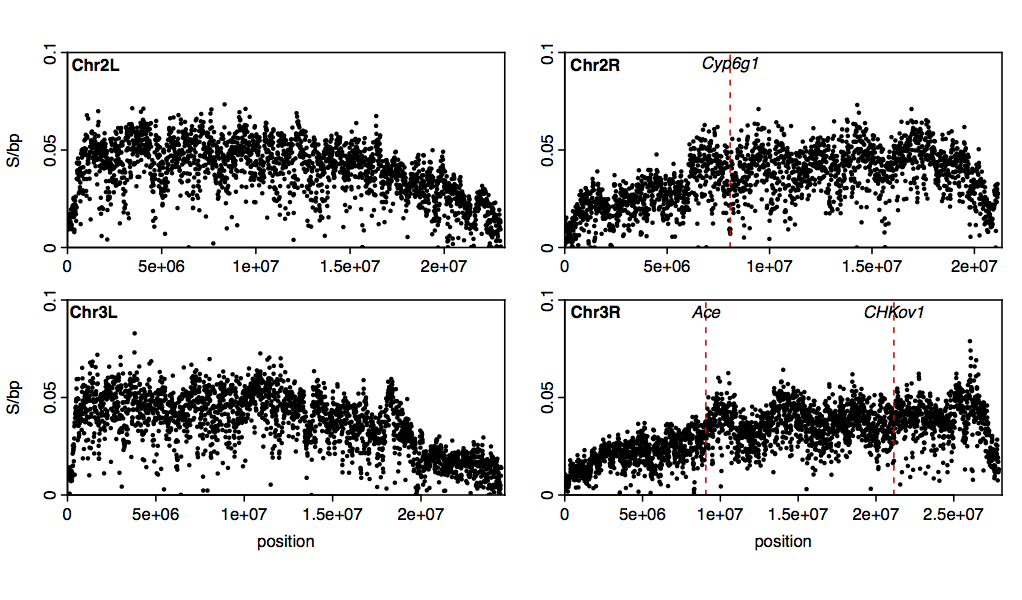

Supplement: S2 Fig — Each point represents the mean S/bp value in a 10Kb window. Red vertical lines indicate the positions of the positive controls, Ace, Cyp6g1, and CHKov1. (TIF) [file pgen.1009373.s002.tif]

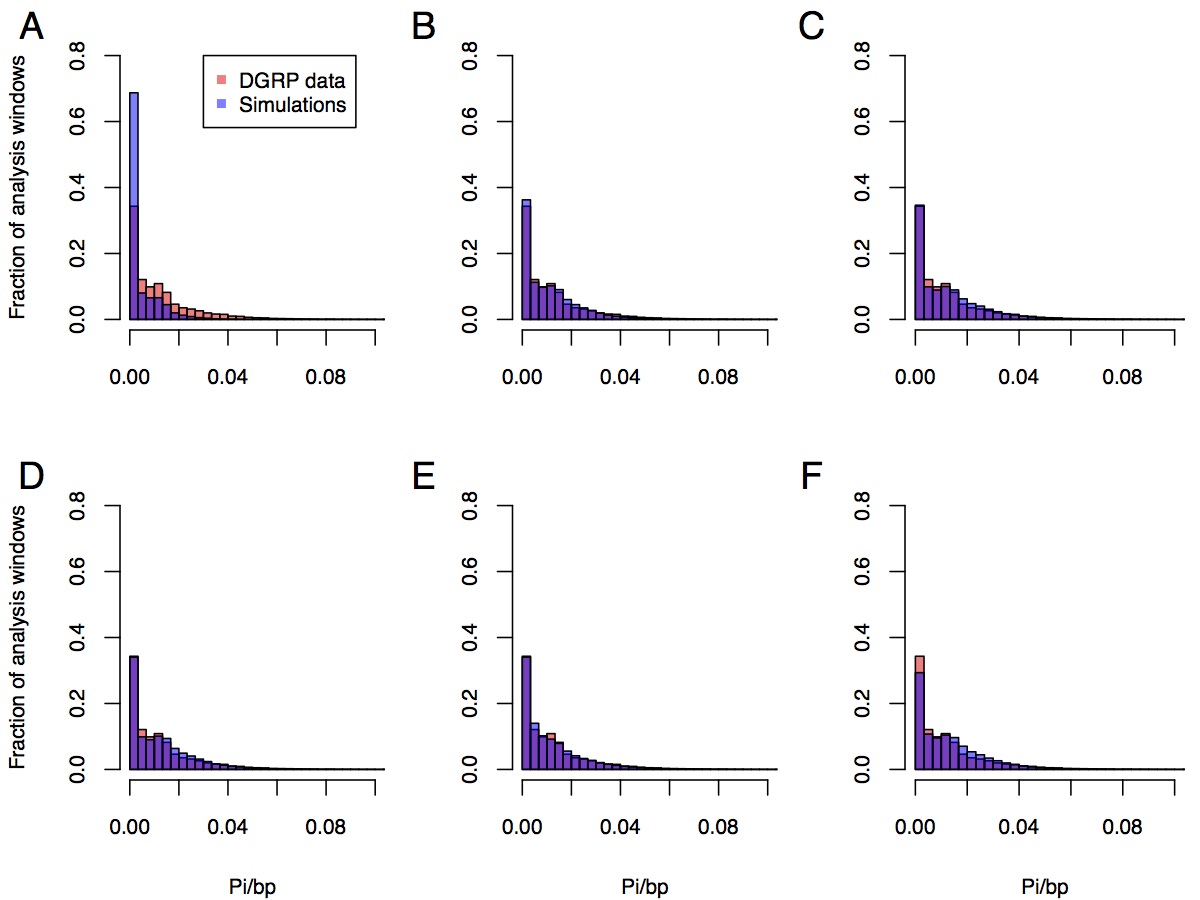

Supplement: S3 Fig — 2015 [34]. The distribution of Pi/bp computed in short introns of length 10bps or longer in DGRP data is compared with Pi/bp values computed in a range of simulated neutral demographic models tested in Garud et al. 2015. The models tested are as follows: (A) a constant Ne = 106 model, (B) a constant Ne = 2.7x10^6^ model, (C) a severe short bottleneck model, (D) a shallow long bottleneck model, (E) the implemented admixture model in Garud et al. 2015 [34], and (F) the implemented admixture + bottleneck model in Garud et al. 2015 [34]. Short intron lengths matching those in data were used were used in simulations. Each simulation contains10x the number of short intron fragments as observed in the data. S6 Fig shows the fit of these distributions in quantile-quantile plots. (TIF) [file pgen.1009373.s003.tif]

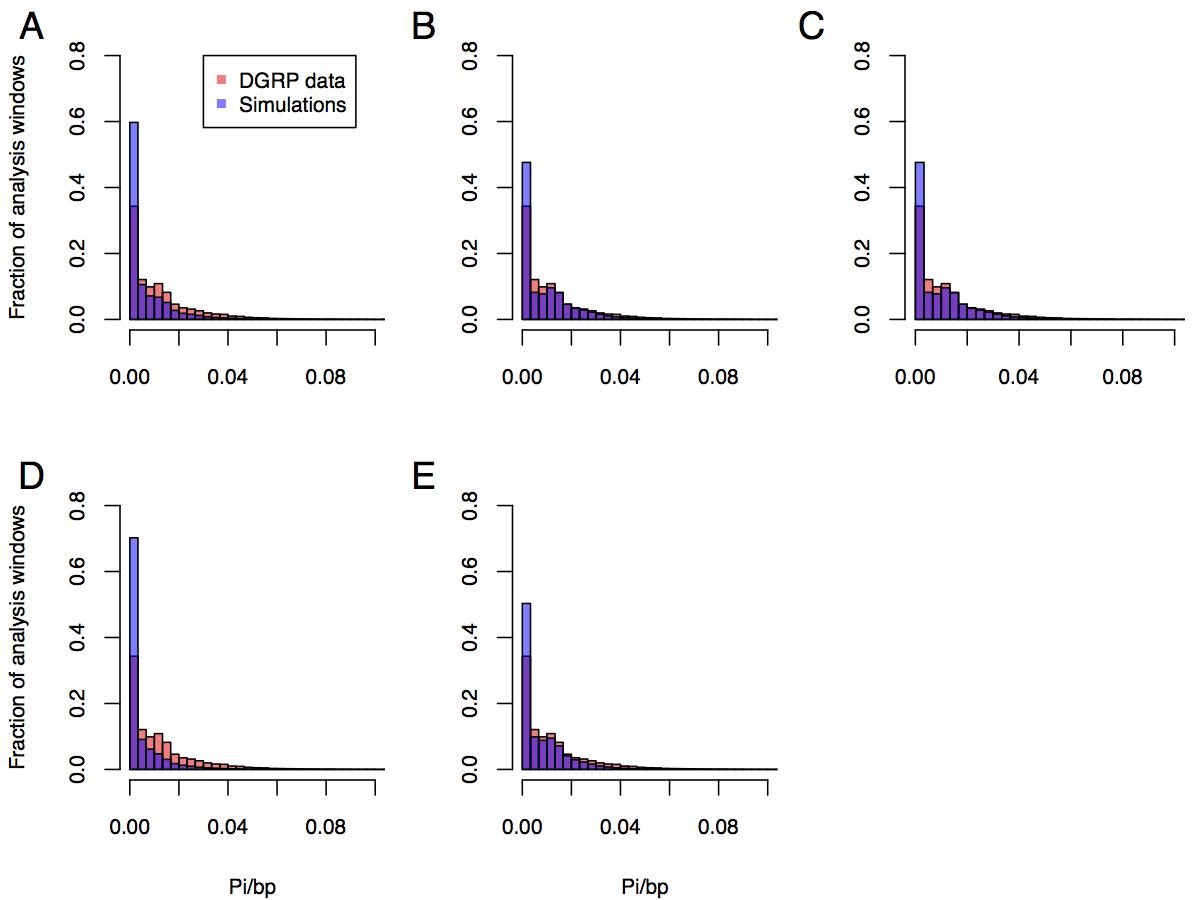

Supplement: S4 Fig — [50], Harris et al. [45], and Arguello et al [51]. The distribution of Pi/bp computed in short introns of length 10bps or longer in DGRP data is compared with Pi/bp values computed in a range of simulated neutral demographic models from Duchen et al. [50], Harris et al. [45], and Arguello et al. [51]. The models tested are as follows: (A) The admixture model proposed by Duchen et al. 2013 [50], simulated with parameter values corresponding to the mode of the posterior. (B) The admixture + bottleneck model proposed by Duchen et al. 2013 [50], simulated with parameter values corresponding to the mode of the posterior. (C) The admixture model proposed by Duchen et al. 2013 [50], simulated with parameter values drawn from the posterior distribution. (D) The implemented admixture model in Harris et al. 2018 [45], simulated with parameter values drawn from the posterior distribution. (E) The admixture model proposed by Arguello et al. 2019 [51]. Short intron lengths matching those in data were used were used in simulations. Each simulation contains10x the number of short intron fragments as observed in the data. S7 Fig shows the fit of these distributions in quantile-quantile plots. (TIF) [file pgen.1009373.s004.tif]

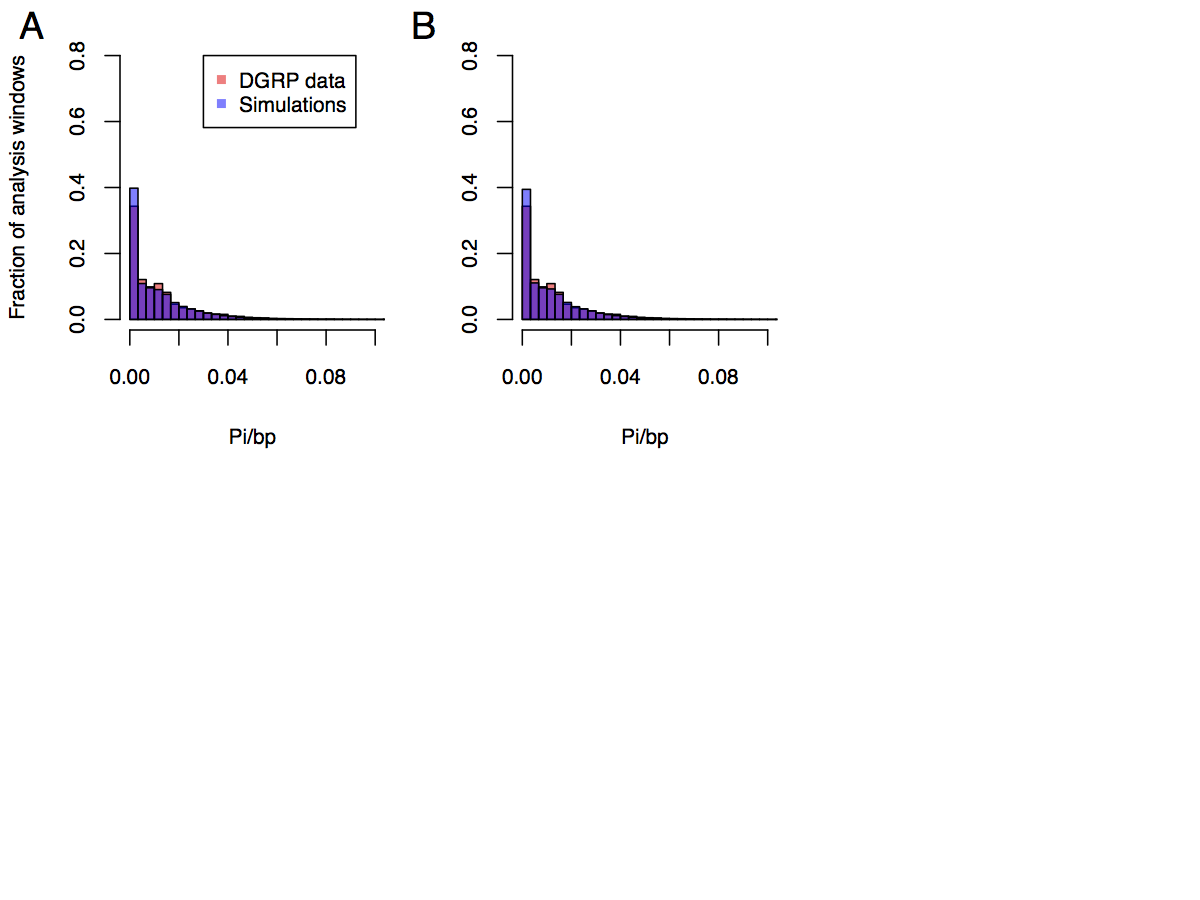

Supplement: S5 Fig — The distribution of Pi/bp computed in short introns of length 10bps or longer in DGRP data is compared with Pi/bp values computed in two demographic models inferred in this paper to fit the DGRP (Models presented in Fig 3J and 3K) (A) A variant of the Duchen et al. 2013 [50] admixture model where North America, Europe, and Africa have fixed population sizes. North American population size = 1.11x10^6, European population size = .7x10^6, and African population size held constant at the value inferred in Duchen et al. 2013 [50]. (B) A variant of the Duchen et al. 2013 [50] admixture model where North America, Europe, and Africa have fixed population sizes. North American population size = 1.6x10^6, European population size = .7x10^6, and African population size held constant at the value inferred in Duchen et al. 2013 [50]. Short intron lengths matching those in data were used were used in simulations. Each simulation contains10x the number of short intron fragments as observed in the data. S8 Fig shows the fit of these distributions in quantile-quantile plots. (TIF) [file pgen.1009373.s005.tif]

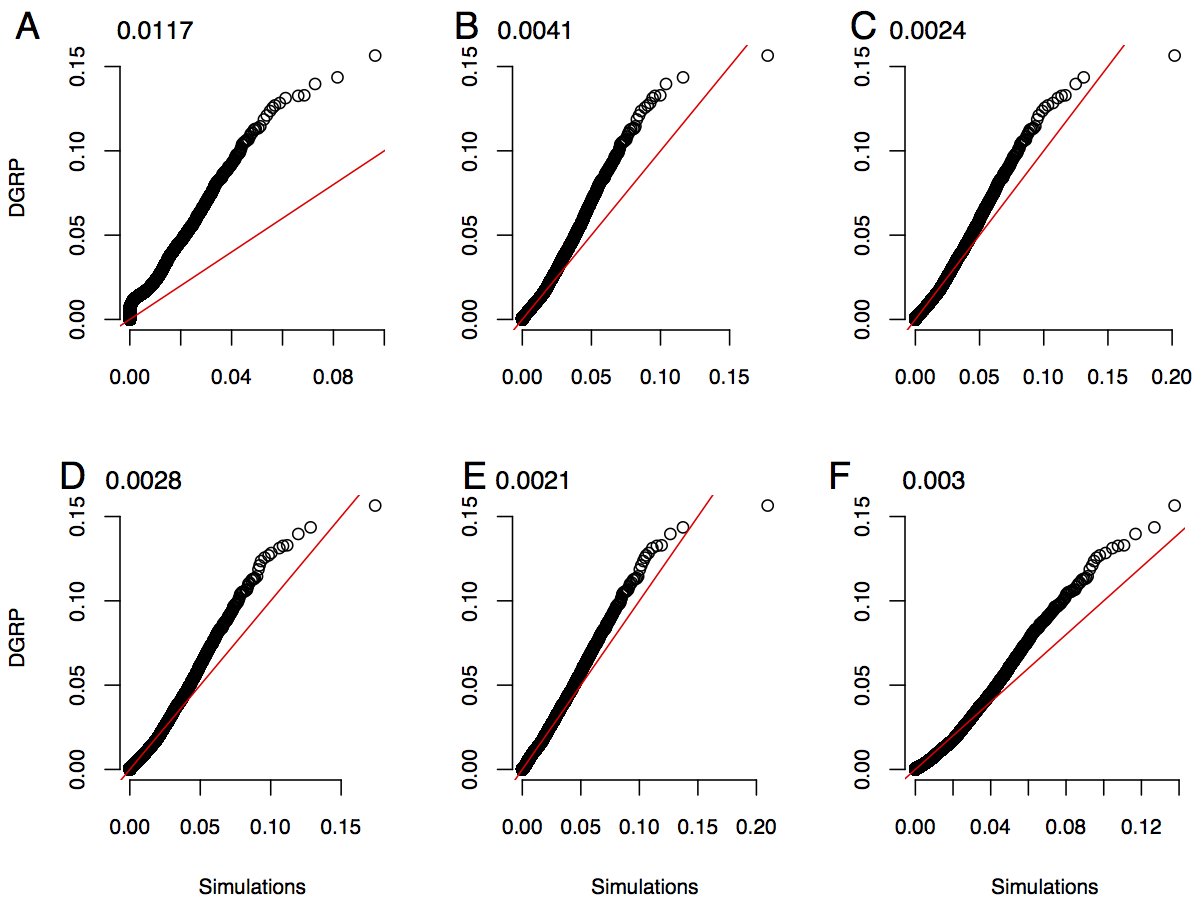

Supplement: S6 Fig — 2015 [34]. These plots quantify the fit of the distributions plotted in S3 Fig. The distribution of Pi/bp computed in short introns of length 10bps or longer in DGRP data is compared with Pi values computed in a range of simulated neutral demographic models tested in Garud et al. 2015 [34]. The models tested are as follows: (A) a constant Ne = 106 model, (B) a constant Ne = 2.7x10^6^ model, (C) a severe short bottleneck model, (D) a shallow long bottleneck model, (E) the implemented admixture model in Garud et al. 2015 [34], and (F) the implemented admixture + bottleneck model in Garud et al. 2015 [34]. The root mean square error (RMSE) of the fit is reported in the top left corner of each plot. (TIF) [file pgen.1009373.s006.tif]

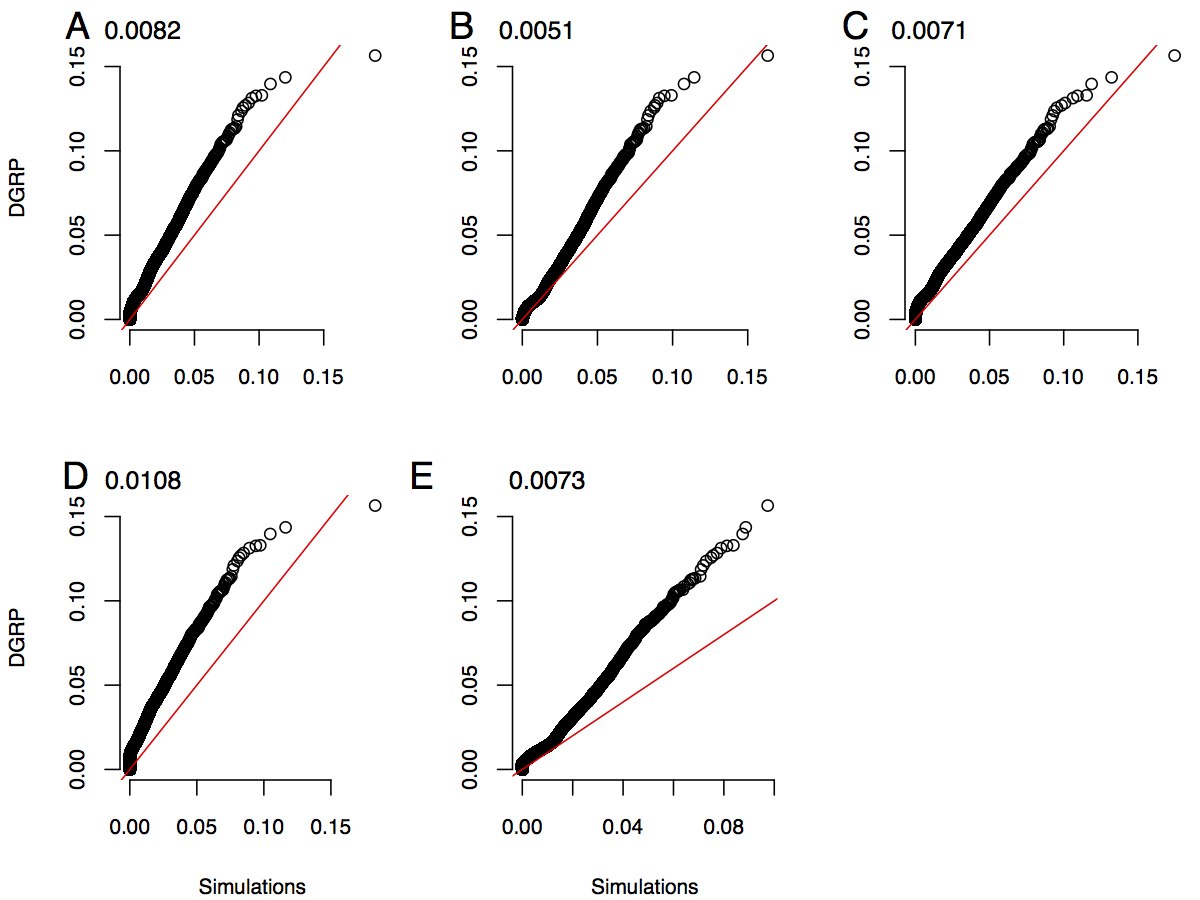

Supplement: S7 Fig — [50], Harris et al. [45], and Arguello et al [51]. These plots quantify the fit of the distributions plotted in S4 Fig. The distribution of Pi/bp computed in short introns of length 10bps or longer in DGRP data is compared with Pi/bp values computed in a range of simulated neutral demographic models from Duchen et al. [50], Harris et al. [45], and Arguello et al. [51]. The models tested are as follows: (A) The admixture model proposed by Duchen et al. 2013 [50], simulated with parameter values corresponding to the mode of the posterior. (B) The admixture + bottleneck model proposed by Duchen et al. 2013 [50], simulated with parameter values corresponding to the mode of the posterior. (C) The admixture model proposed by Duchen et al. 2013 [50], simulated with parameter values drawn from the posterior distribution. (D) The implemented admixture model in Harris et al. 2018 [45], simulated with parameter values drawn from the posterior distribution. (E) The admixture model proposed by Arguello et al. 2019 [51]. The root mean square error (RMSE) of the fit is reported in the top left corner of each plot. (TIF) [file pgen.1009373.s007.tif]

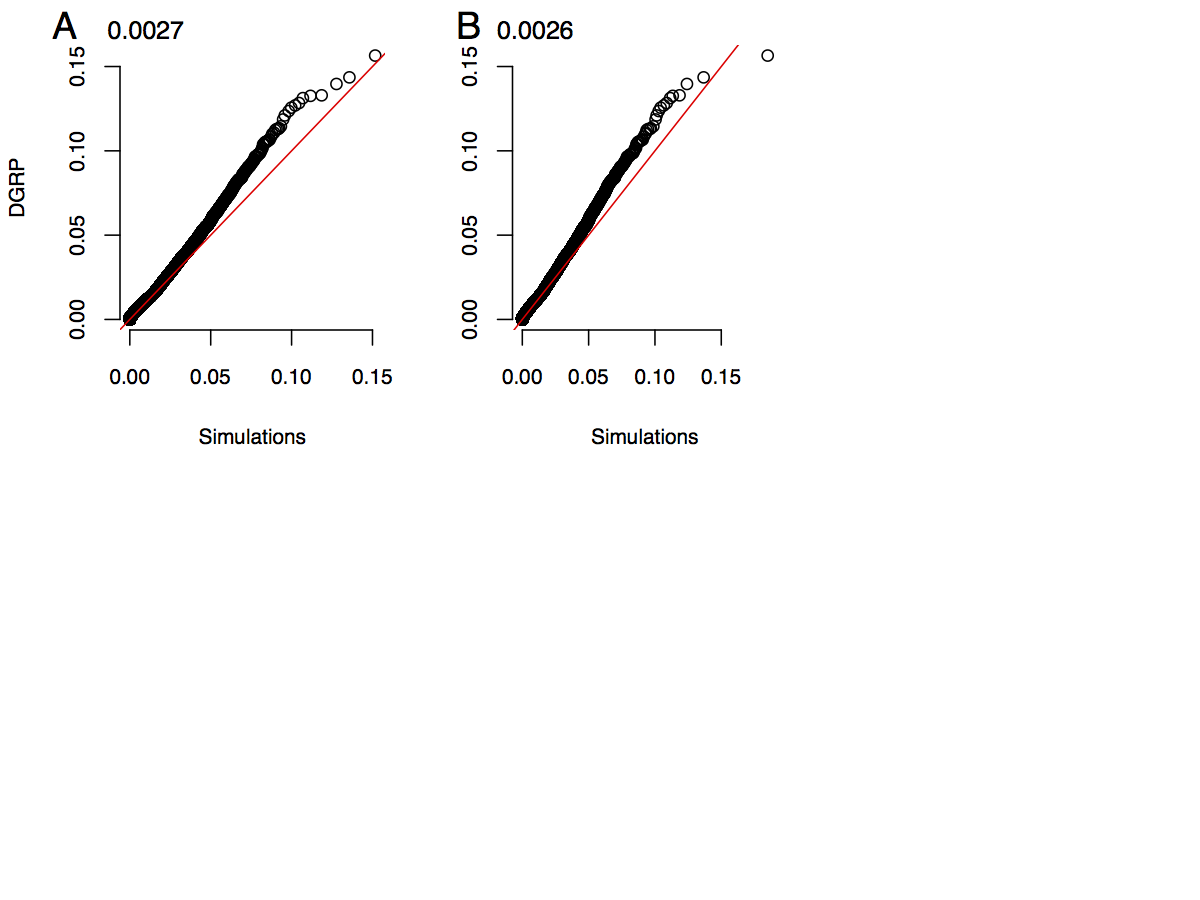

Supplement: S8 Fig — These plots quantify the fit of the distributions plotted in S6 Fig. The distribution of Pi/bp computed in short introns of length 10bps or longer in DGRP data is compared with Pi/bp values computed in two demographic models inferred in this paper to fit the DGRP (Models presented in Fig 3J and 3K) (A) A variant of the Duchen et al. 2013 [50] admixture model where North America, Europe, and Africa have fixed population sizes. North American population size = 1.11x10^6, European population size = .7x10^6, and African population size held constant at the value inferred in Duchen et al. 2013 [50]. (B) A variant of the Duchen et al. 2013 [50] admixture model where North America, Europe, and Africa have fixed population sizes. North American population size = 1.6x10^6, European population size = .7x10^6, and African population size held constant at the value inferred in Duchen et al. 2013 [50]. The root mean square error (RMSE) of the fit is reported in the top left corner of each plot. (TIF) [file pgen.1009373.s008.tif]

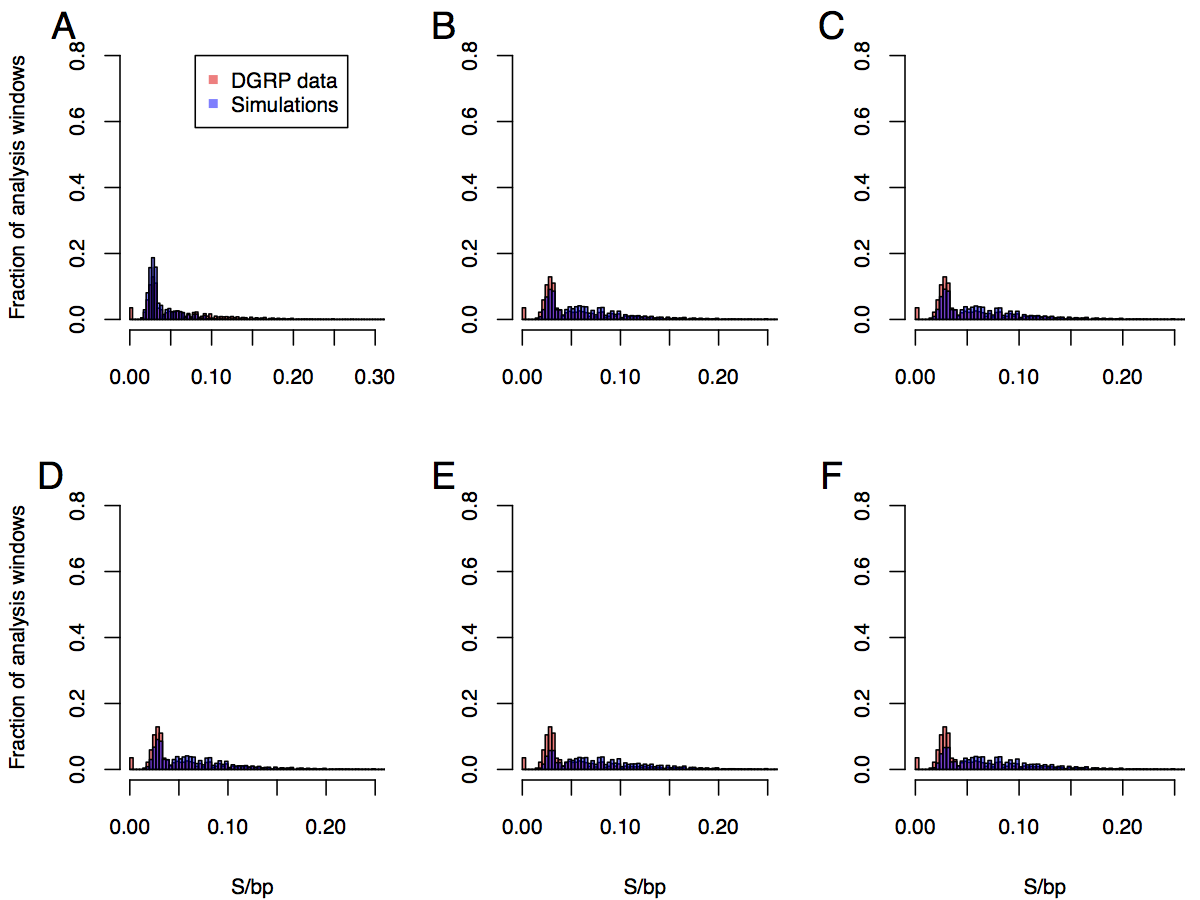

Supplement: S9 Fig — 2015 [34]. The distribution of S/bp computed in short introns of length 10bps or longer in DGRP data is compared with S/bp values computed in a range of simulated neutral demographic models tested in Garud et al. 2015. The models tested are as follows: (A) a constant Ne = 106 model, (B) a constant Ne = 2.7x10^6^ model, (C) a severe short bottleneck model, (D) a shallow long bottleneck model, (E) the implemented admixture model in Garud et al. 2015, and (F) the implemented admixture + bottleneck model in Garud et al. 2015. Short intron lengths matching those in data were used were used in simulations. Each simulation contains10x the number of short intron fragments as observed in the data. S12 Fig shows the fit of these distributions in quantile-quantile plots. (TIF) [file pgen.1009373.s009.tif]

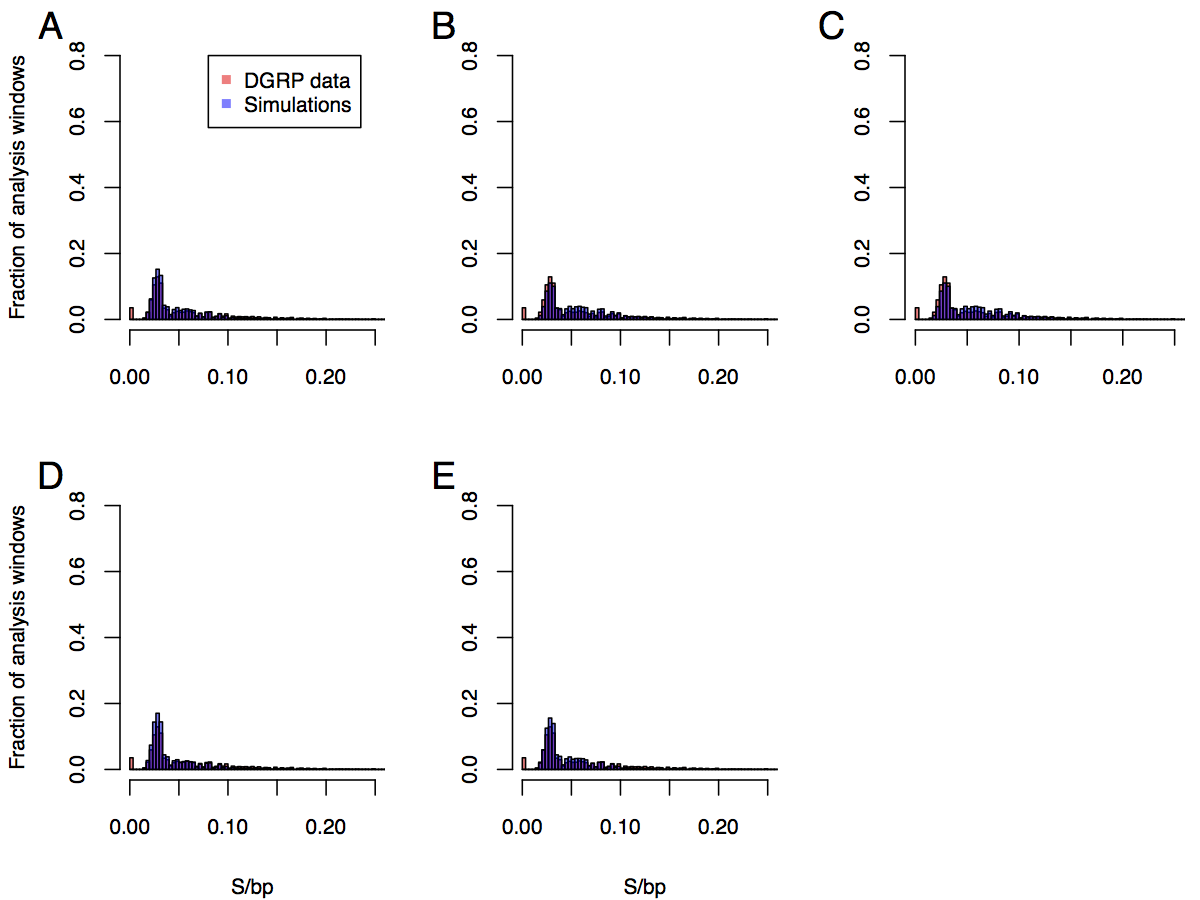

Supplement: S10 Fig — [50], Harris et al. [45], and Arguello et al [51]. The distribution of S/bp computed in short introns of length 10bps or longer in DGRP data is compared with S/bp values computed in a range of simulated neutral demographic models from Duchen et al. [50], Harris et al. [45], and Arguello et al. [51]. The models tested are as follows: (A) The admixture model proposed by Duchen et al. 2013 [50], simulated with parameter values corresponding to the mode of the posterior. (B) The admixture + bottleneck model proposed by Duchen et al. 2013 [50], simulated with parameter values corresponding to the mode of the posterior. (C) The admixture model proposed by Duchen et al. 2013 [50], simulated with parameter values drawn from the posterior distribution. (D) The implemented admixture model in Harris et al. 2018 [45], simulated with parameter values drawn from the posterior distribution. (E) The admixture model proposed by Arguello et al. 2019 [51]. Short intron lengths matching those in data were used were used in simulations. Each simulation contains10x the number of short intron fragments as observed in the data. S13 Fig shows the fit of these distributions in quantile-quantile plots. (TIF) [file pgen.1009373.s010.tif]

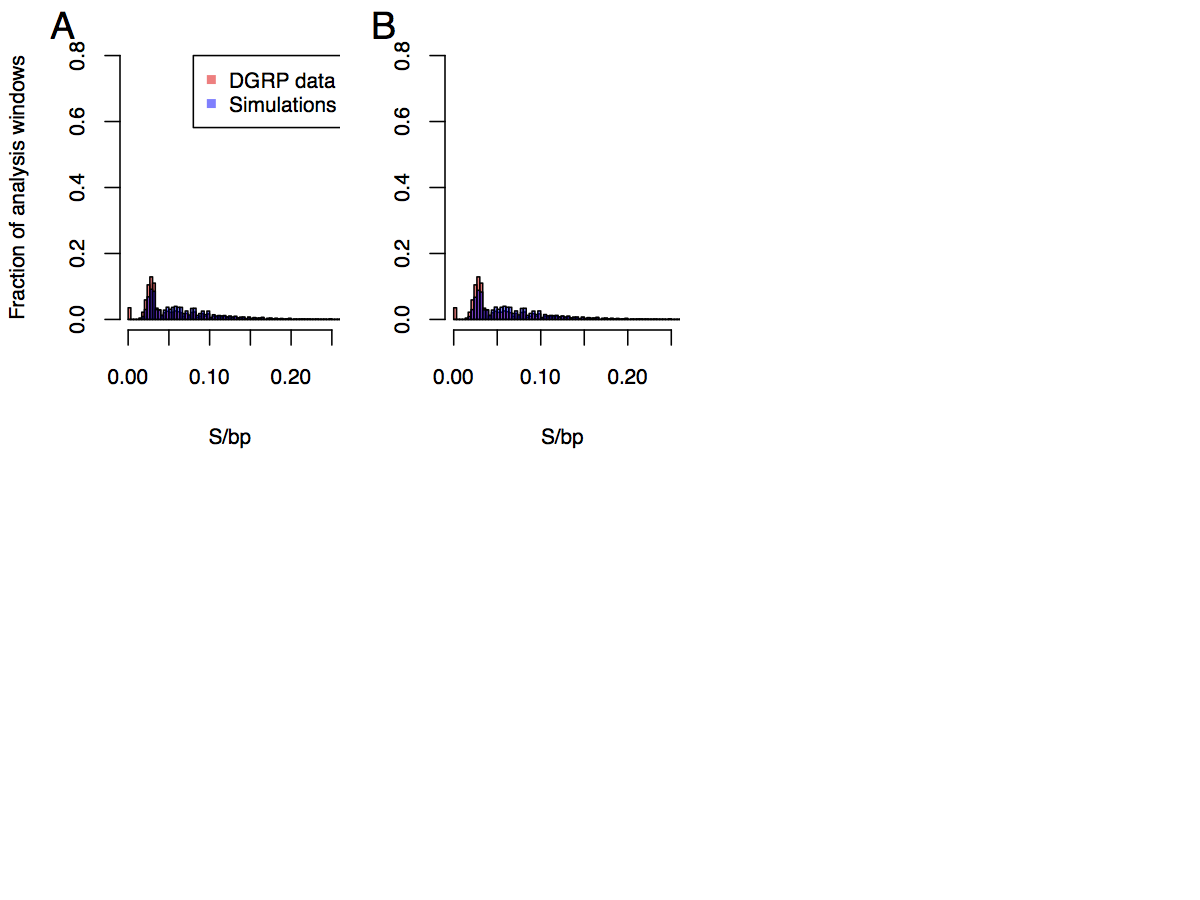

Supplement: S11 Fig — The distribution of S/bp computed in short introns of length 10bps or longer in DGRP data is compared with S/bp values computed in two demographic models inferred in this paper to fit the DGRP (Models presented in Fig 3J and 3K) (A) A variant of the Duchen et al. 2013 [50] admixture model where North America, Europe, and Africa have fixed population sizes. North American population size = 1.11x10^6, European population size = .7x10^6, and African population size held constant at the value inferred in Duchen et al. 2013 [50]. (B) A variant of the Duchen et al. 2013 [50] admixture model where North America, Europe, and Africa have fixed population sizes. North American population size = 1.6x10^6, European population size = .7x10^6, and African population size held constant at the value inferred in Duchen et al. 2013 [50]. Short intron lengths matching those in data were used were used in simulations. Each simulation contains10x the number of short intron fragments as observed in the data. S8 Fig shows the fit of these distributions in quantile-quantile plots. (TIF) [file pgen.1009373.s011.tif]

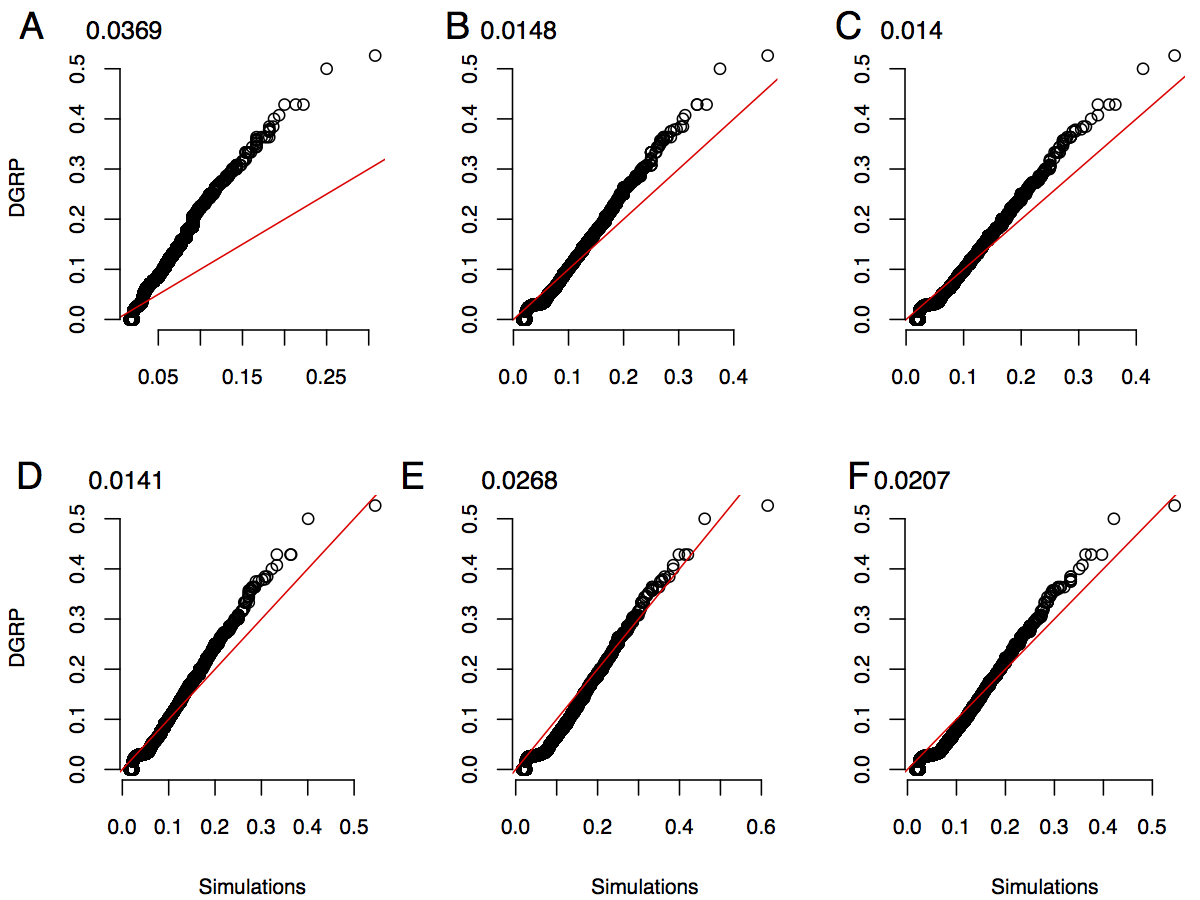

Supplement: S12 Fig — 2015 [34]. These plots quantify the fit of the distributions plotted in S9 Fig. The distribution of S/bp computed in short introns of length 10bps or longer in DGRP data is compared with S/bp values computed in a range of simulated neutral demographic models tested in Garud et al. 2015 [34]. The models tested are as follows: (A) a constant Ne = 106 model, (B) a constant Ne = 2.7x10^6^ model, (C) a severe short bottleneck model, (D) a shallow long bottleneck model, (E) the implemented admixture model in Garud et al. 2015 [34], and (F) the implemented admixture + bottleneck model in Garud et al. 2015 [34]. The root mean square error (RMSE) of the fit is reported in the top left corner of each plot. (TIF) [file pgen.1009373.s012.tif]

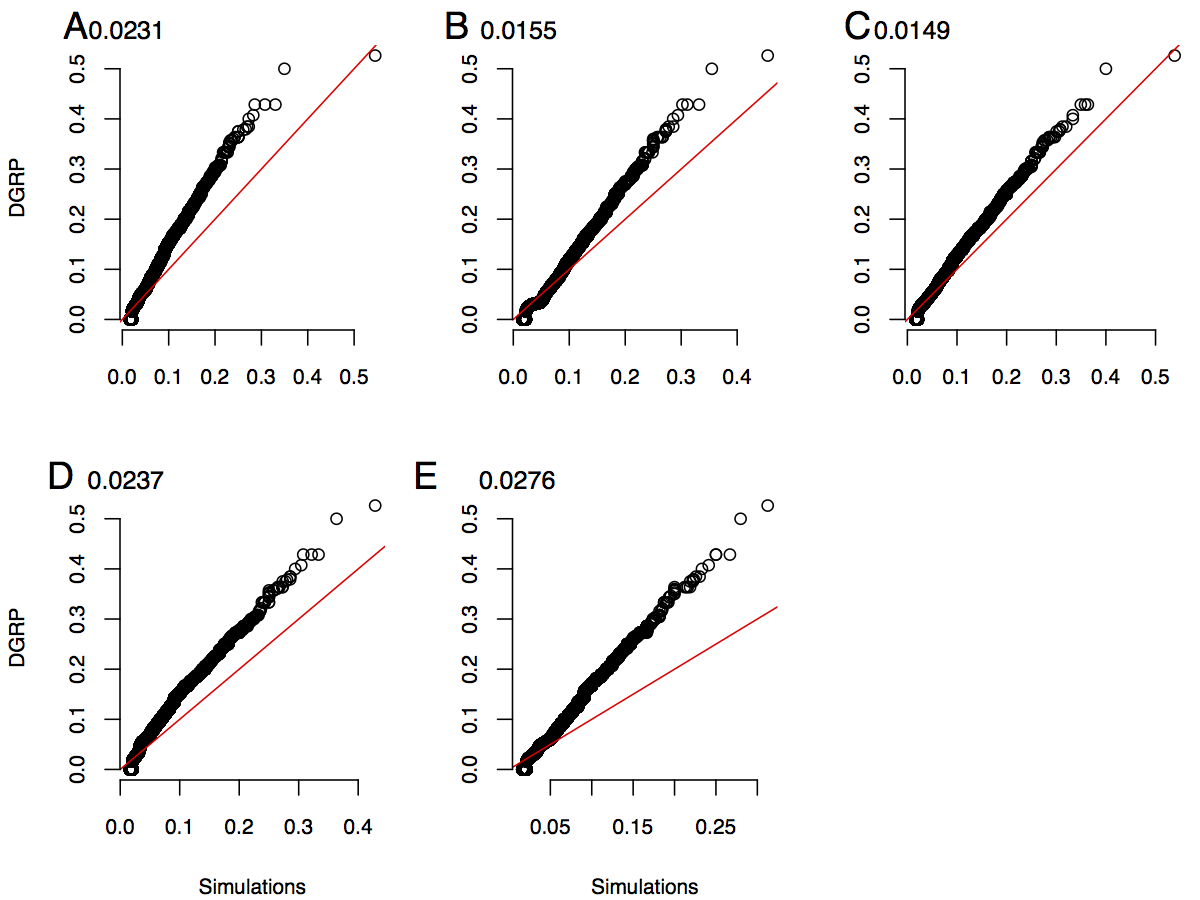

Supplement: S13 Fig — [50], Harris et al. [45], and Arguello et al [51]. These plots quantify the fit of the distributions plotted in S10 Fig. The distribution of S/bp computed in short introns of length 10bps or longer in DGRP data is compared with S/bp values computed in a range of simulated neutral demographic models from Duchen et al. [50], Harris et al. [45], and Arguello et al. [51]. The models tested are as follows: (A) The admixture model proposed by Duchen et al. 2013 [50], simulated with parameter values corresponding to the mode of the posterior. (B) The admixture + bottleneck model proposed by Duchen et al. 2013 [50], simulated with parameter values corresponding to the mode of the posterior. (C) The admixture model proposed by Duchen et al. 2013 [50], simulated with parameter values drawn from the posterior distribution. (D) The implemented admixture model in Harris et al. 2018 [45], simulated with parameter values drawn from the posterior distribution. (E) The admixture model proposed by Arguello et al. 2019 [51]. The root mean square error (RMSE) of the fit is reported in the top left corner of each plot. (TIF) [file pgen.1009373.s013.tif]

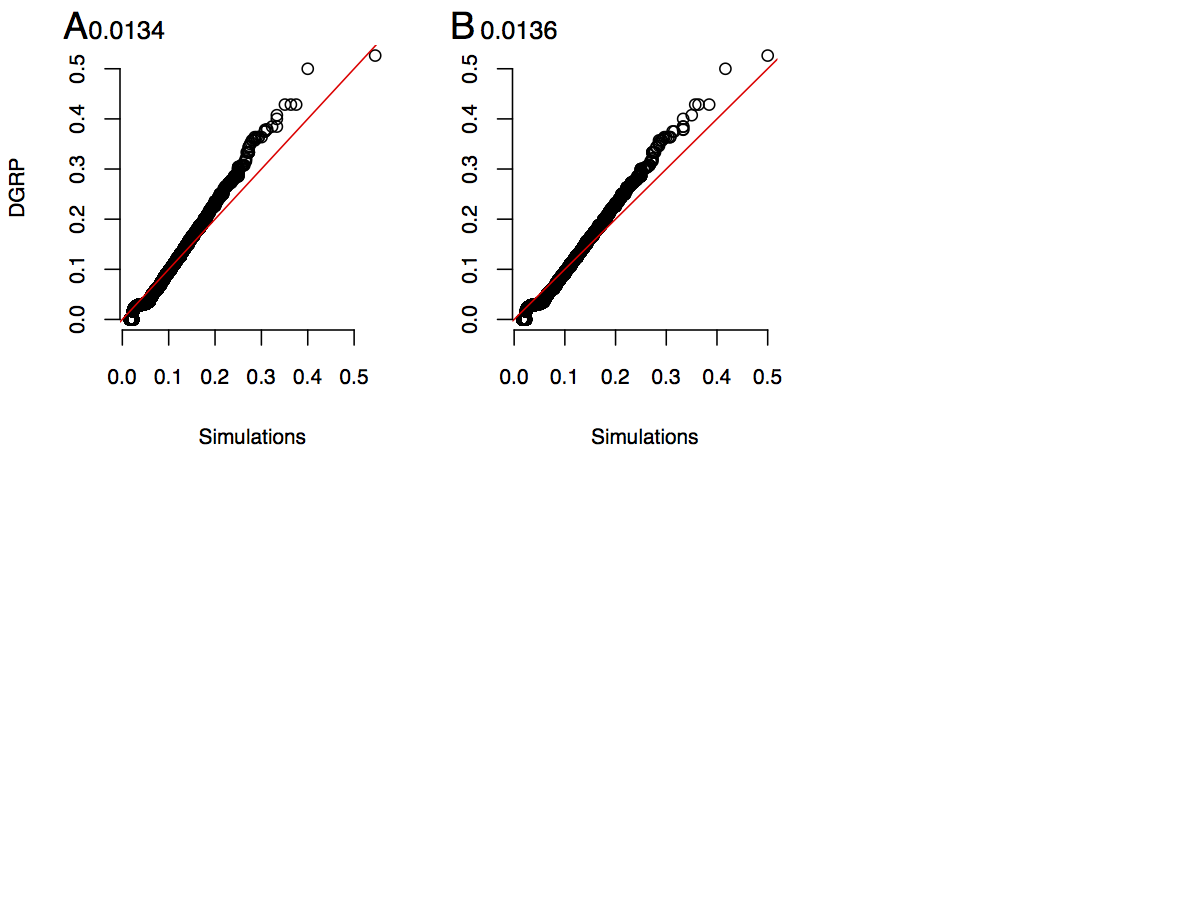

Supplement: S14 Fig — These plots quantify the fit of the distributions plotted in S11 Fig. The distribution of S/bp computed in short introns of length 10bps or longer in DGRP data is compared with S/bp values computed in two demographic models inferred in this paper to fit the DGRP (Models presented in Fig 3J and 3K) (A) A variant of the Duchen et al. 2013 [50] admixture model where North America, Europe, and Africa have fixed population sizes. North American population size = 1.11x10^6, European population size = .7x10^6, and African population size held constant at the value inferred in Duchen et al. 2013 [50]. (B) A variant of the Duchen et al. 2013 [50] admixture model where North America, Europe, and Africa have fixed population sizes. North American population size = 1.6x10^6, European population size = .7x10^6, and African population size held constant at the value inferred in Duchen et al. 2013 [50]. The root mean square error (RMSE) of the fit is reported in the top left corner of each plot. (TIF) [file pgen.1009373.s014.tif]

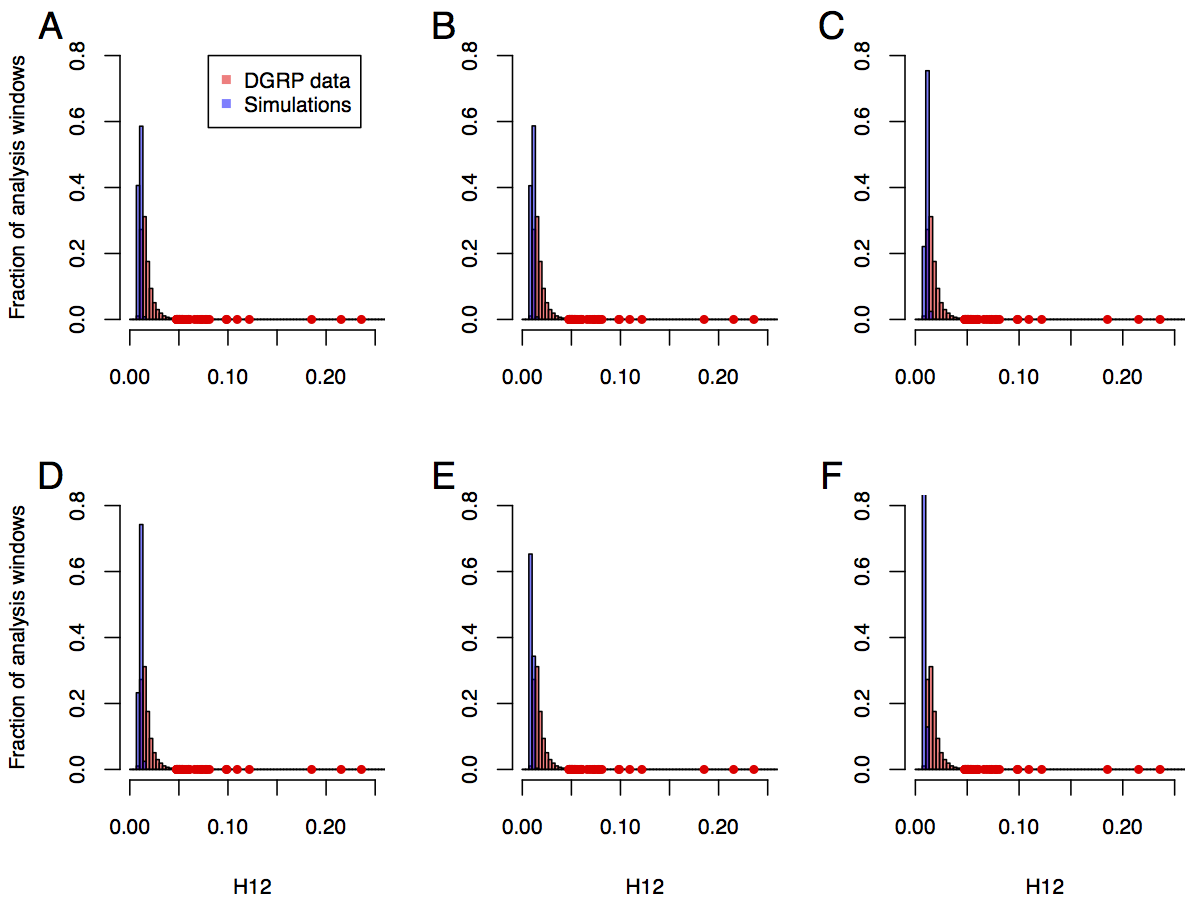

Supplement: S15 Fig — 2015 [34]. The DGRP H12 values are compared with H12 values computed in a range of simulated neutral demographic models from Garud et al. 2015. The models tested are as follows: (A) a constant Ne = 106 model, (B) a constant Ne = 2.7x10^6^ model, (C) a severe short bottleneck model, (D) a shallow long bottleneck model, (E) the implemented admixture model in Garud et al. 2015 [34], and (F) the implemented admixture + bottleneck model in Garud et al. 2015. The number of analysis windows generated for the simulated models (n = 69,113) equals the number of analysis windows for the DGRP data, after excluding regions of low recombination rates. The red points indicate the H12 values for the top 50 peaks in the DGRP data. (TIF) [file pgen.1009373.s015.tif]

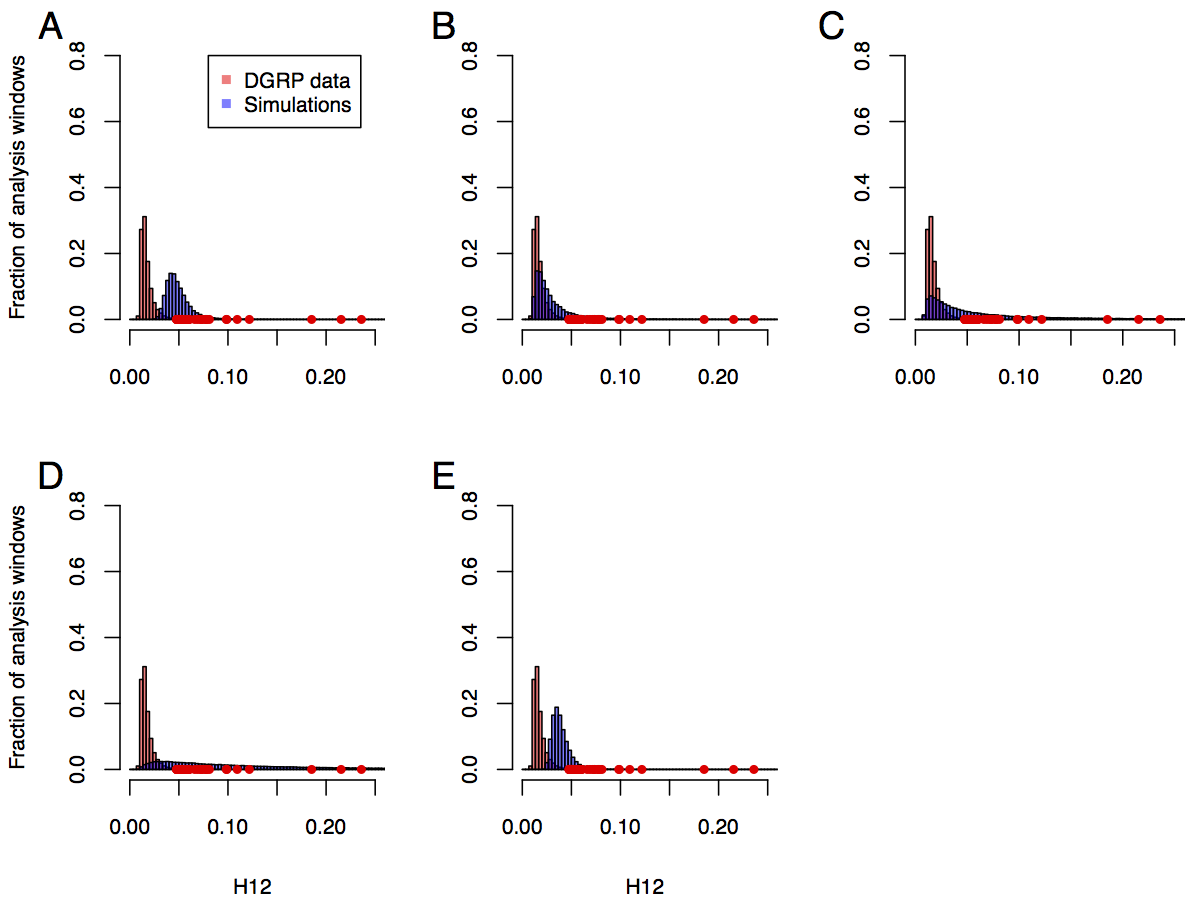

Supplement: S16 Fig — [50], Harris et al. [45], and Arguello et al [51]. The DGRP H12 values are compared with H12 values computed in a range of simulated neutral demographic models from Duchen et al. [50], Harris et al. [45], and Arguello et al. [51]. The models tested are as follows: (A) The admixture model proposed by Duchen et al. 2013 [50], simulated with parameter values corresponding to the mode of the posterior. (B) The admixture + bottleneck model proposed by Duchen et al. 2013 [50], simulated with parameter values corresponding to the mode of the posterior. (C) The admixture model proposed by Duchen et al. 2013 [50], simulated with parameter values drawn from the posterior distribution. (D) The implemented admixture model in Harris et al. 2018 [45], simulated with parameter values drawn from the posterior distribution. (E) The admixture model proposed by Arguello et al. 2019 [51]. The number of analysis windows generated for the simulated models (n = 69,113) equals the number of analysis windows for the DGRP data, after excluding regions of low recombination rates. The red points indicate the H12 values for the top 50 peaks in the DGRP data. (TIF) [file pgen.1009373.s016.tif]

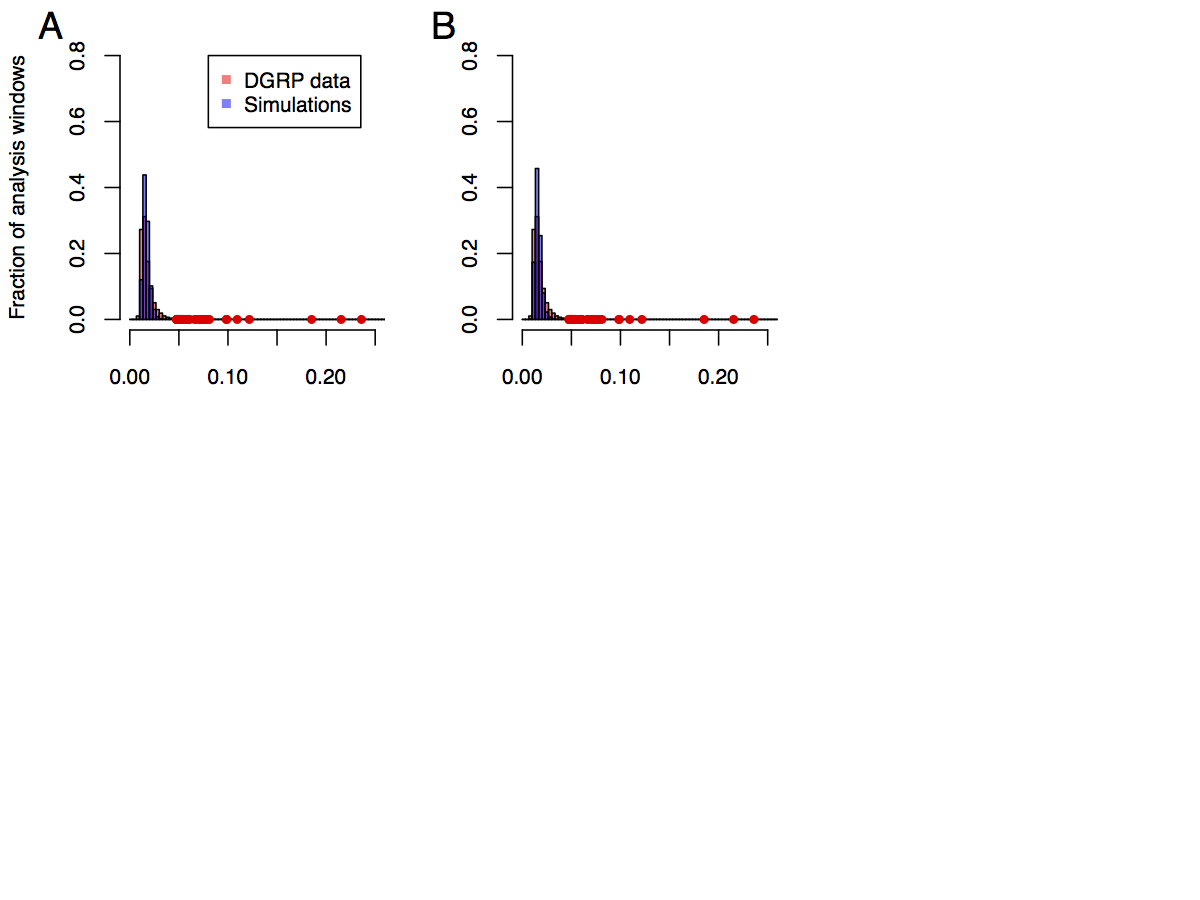

Supplement: S17 Fig — The two models are depicted in Fig 3J and 3K and are (A) A variant of the Duchen et al. 2013 [50] admixture model where North America, Europe, and Africa have fixed population sizes. North American population size = 1.11x10^6, European population size = .7x10^6, and African population size held constant at the value inferred in Duchen et al. 2013 [50]. (B) A variant of the Duchen et al. 2013 [50] admixture model where North America, Europe, and Africa have fixed population sizes. North American population size = 1.6x10^6, European population size = .7x10^6, and African population size held constant at the value inferred in Duchen et al. 2013 [50]. The number of analysis windows generated for the simulated models (n = 69,113) equals the number of analysis windows for the DGRP data, after excluding regions of low recombination rates. The red points indicate the H12 values for the top 50 peaks in the DGRP data. (TIF) [file pgen.1009373.s017.tif]

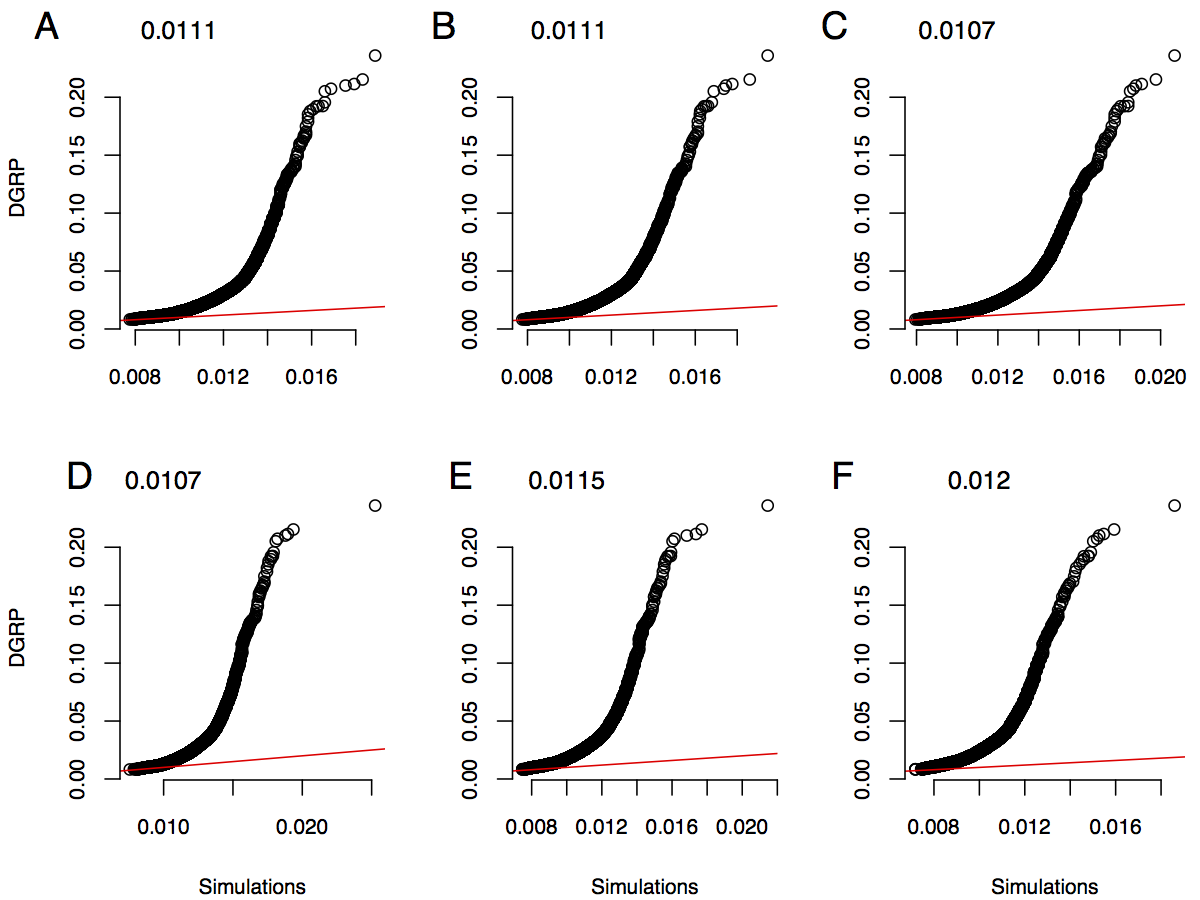

Supplement: S18 Fig — 2015 [34]. These plots quantify the fit of the distributions plotted in S15 Fig. The models tested are as follows: (A) a constant Ne = 106 model, (B) a constant Ne = 2.7x10^6^ model, (C) a severe short bottleneck model, (D) a shallow long bottleneck model, (E) the implemented admixture model in Garud et al. 2015 [34], and (F) the implemented admixture + bottleneck model in Garud et al. 2015 [34]. The root mean square error (RMSE) of the fit is reported in the top left corner of each plot. (TIF) [file pgen.1009373.s018.tif]

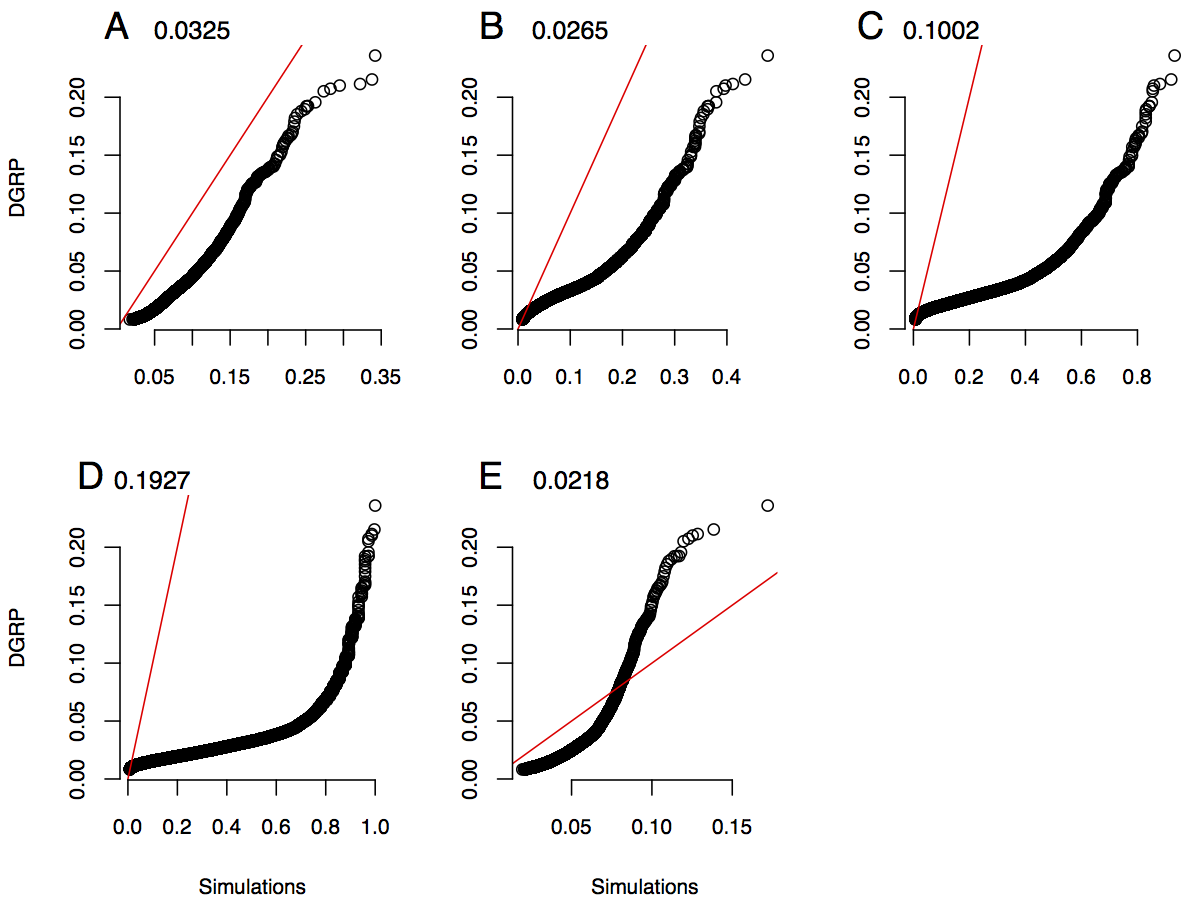

Supplement: S19 Fig — [50], Harris et al. [45], and Arguello et al [51]. These plots quantify the fit of the distributions plotted in S16 Fig. The models tested are as follows: (A) The admixture model proposed by Duchen et al. 2013 [50], simulated with parameter values corresponding to the mode of the posterior. (B) The admixture + bottleneck model proposed by Duchen et al. 2013 [50], simulated with parameter values corresponding to the mode of the posterior. (C) The admixture model proposed by Duchen et al. 2013 [50], simulated with parameter values drawn from the posterior distribution. (D) The implemented admixture model in Harris et al. 2018 [45], simulated with parameter values drawn from the posterior distribution. (E) The admixture model proposed by Arguello et al. 2019 [51]. The root mean square error (RMSE) of the fit is reported in the top left corner of each plot. (TIF) [file pgen.1009373.s019.tif]

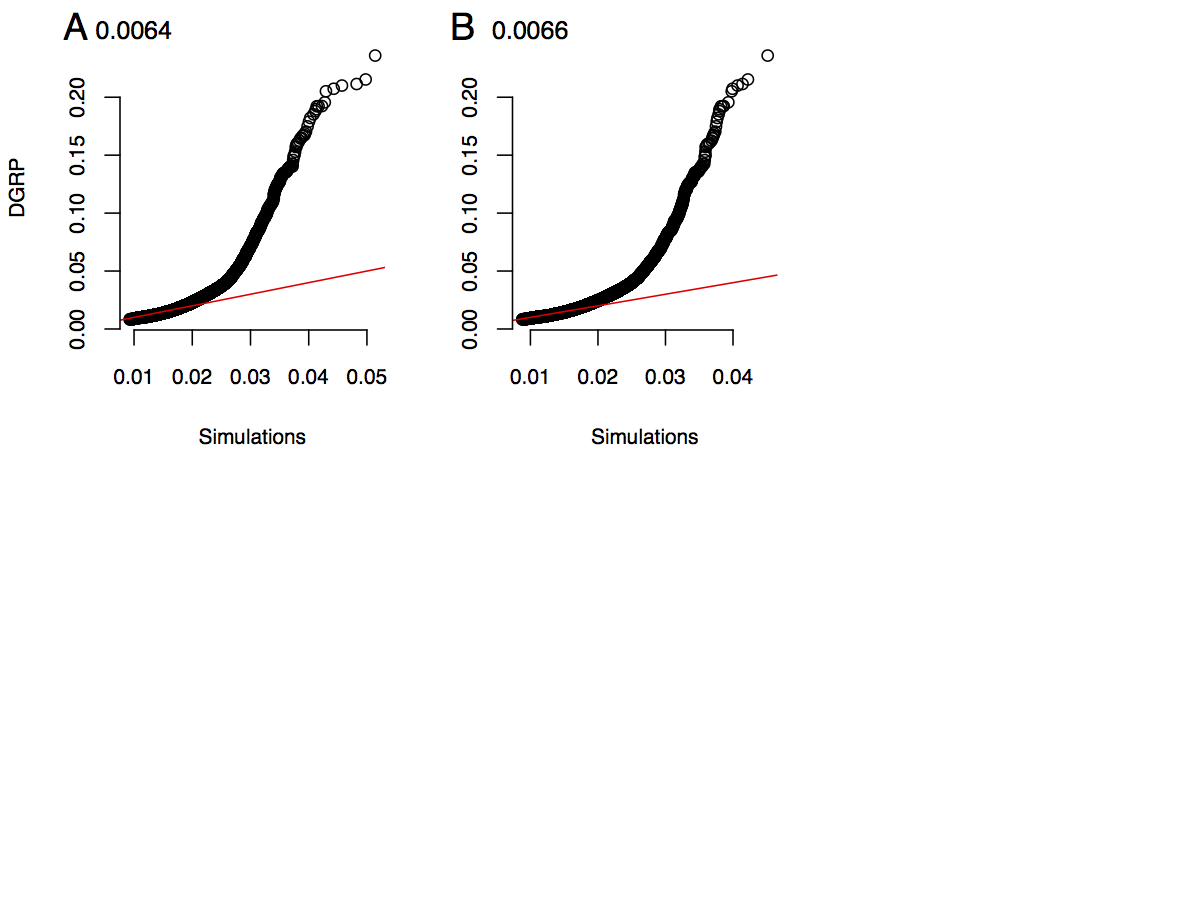

Supplement: S20 Fig — These plots quantify the fit of the distributions plotted in S17 Fig. The models tested are depicted in Fig 3J and 3K (A) A variant of the Duchen et al. 2013 [50] admixture model where North America, Europe, and Africa have fixed population sizes. North American population size = 1.11x10^6, European population size = .7x10^6, and African population size held constant at the value inferred in Duchen et al. 2013 [50]. (B) A variant of the Duchen et al. 2013 [50] admixture model where North America, Europe, and Africa have fixed population sizes. North American population size = 1.6x10^6, European population size = .7x10^6, and African population size held constant at the value inferred in Duchen et al. 2013 [50]. The root mean square error (RMSE) of the fit is reported in the top left corner of each plot. (TIF) [file pgen.1009373.s020.tif]

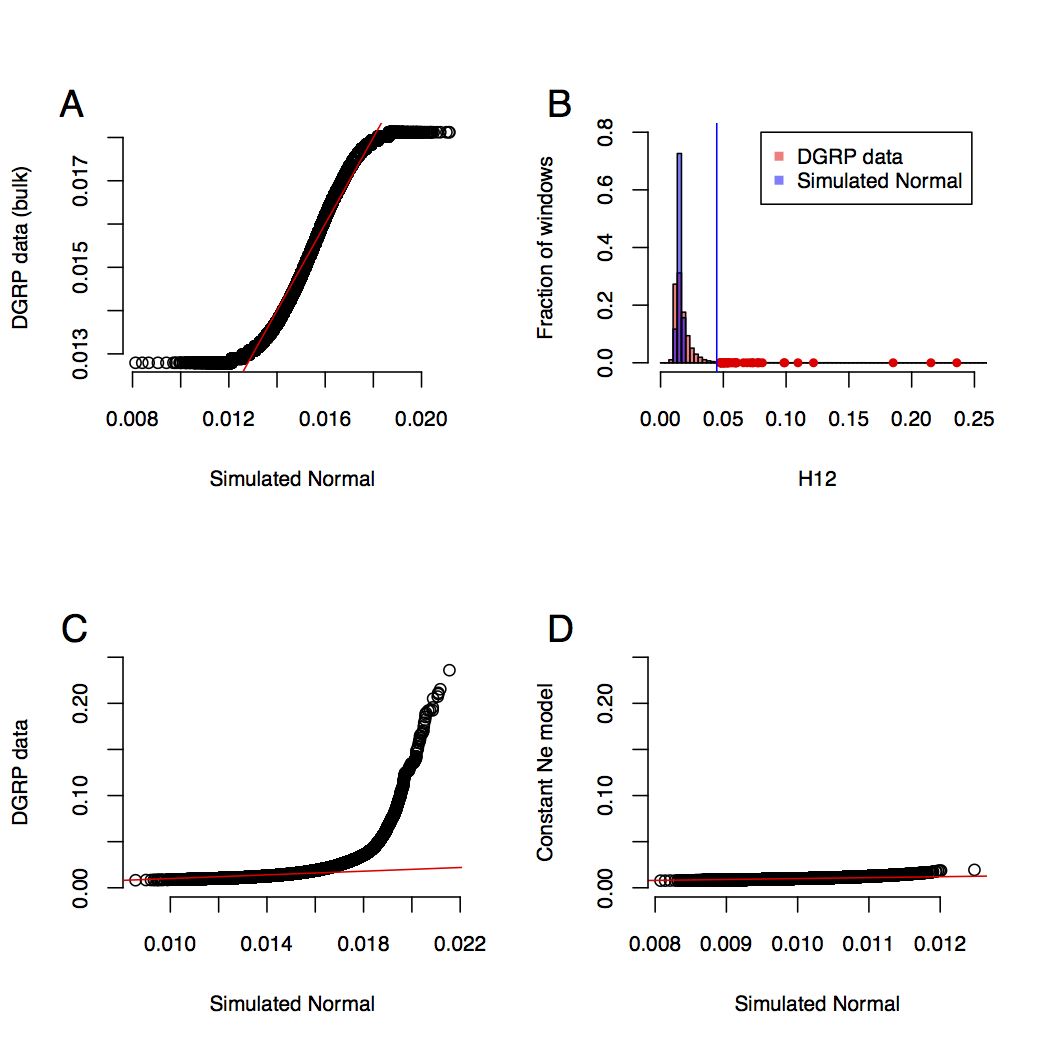

Supplement: S21 Fig — (A) Quantile-quantile plot of H12 values within +/-1 SD of the median value in the DGRP data are compared with a random sample from the fitted Gaussian. The Gaussian was simulated with the mean equalling the median value of H12 in the DGRP data, and the standard deviation estimated from points within 1 standard deviation around the median (Methods). (B) Comparison of distribution of H12 values in DGRP data with that of a simulated Gaussian with a mean and standard deviation from (A). The vertical blue line indicates 11 standard deviations away from the mean of the simulated Gaussian distribution. The red points indicate the H12 values for the top 50 peaks in the DGRP data. (C) QQ-plot of H12 from the entire distribution of DGRP values compared with the same simulated Gaussian from (A). The distribution of H12 values in DGRP data has an extreme elevated tail compared to expectations under a Gaussian (D) QQ-plot comparing H12 values from a constant Ne = 2.7*10^6 model with a Gaussian fitted to its bulk (Methods). Neutral simulations lack the elevated tail present in the data. (TIF) [file pgen.1009373.s021.tif]

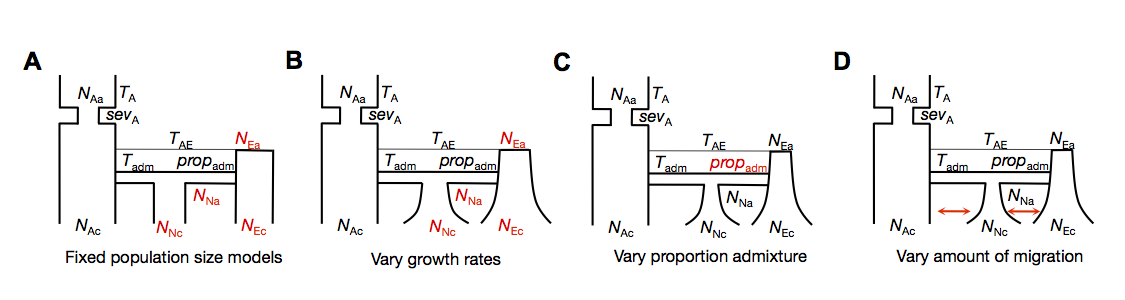

Supplement: S22 Fig — We computed Pi, S, and H12 in variants of the admixture model proposed by Duchen et al. 2013 [50]. The admixture models include: (A) constant population sizes for North America and Europe, (B) different growth rates for North America and Europe, (C) different proportions of admixture, (D) different migration rates. In all cases, the 11 parameters originally inferred by Duchen et al. 2013 [50] were kept constant at the mode of the parameters’ posterior distributions, unless highlighted in red. The parameters highlighted in red were varied. (TIF) [file pgen.1009373.s022.tif]

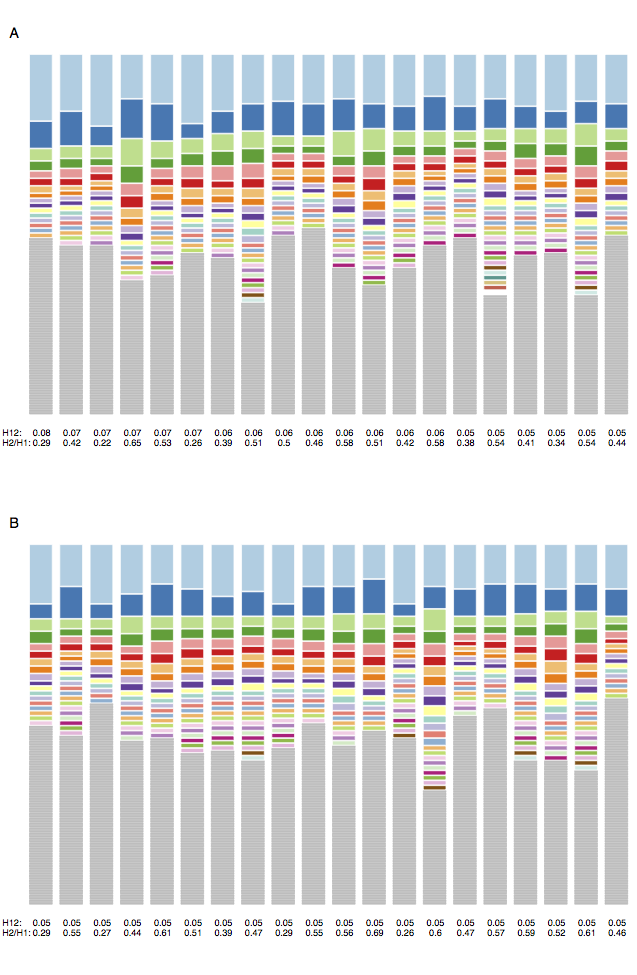

Supplement: S36 Fig — Same as Fig 7, except plotted are haplotype frequency spectra for the (A)11th-30th and the (B) 31st—50th peaks in the DGRP scan. (TIFF) [file pgen.1009373.s036.tiff]
